# In Vivo Evaluation of an Ivermectin and Allicin Combination Treatment for Eradicating Poultry Red Mite

**DOI:** 10.3390/antibiotics12050876

**Published:** 2023-05-09

**Authors:** JeongWoo Kang, MyeongJu Chae, HyunYoung Chae, YongKuk Kwon, JiYoun Lee, Md Akil Hossain

**Affiliations:** 1 Animal Disease Diagnosis Division, Animal and Plant Quarantine Agency (APQA), Ministry of Agriculture, Food and Rural Affairs, 177, Hyeoksin 8-ro, Gimcheon-si 39660, Republic of Korea; hijach@korea.kr (J.K.); iichy33ii@korea.kr (H.C.); 2 Avian Disease Research Division, Animal and Plant Quarantine Agency (APQA), Ministry of Agriculture, Food and Rural Affairs, 177, Hyeoksin 8-ro, Gimcheon-si 39660, Republic of Korea; chaemj@korea.kr (M.C.); kwonyk66@korea.kr (Y.K.); 3 Department of Oral Biology, College of Dentistry, University of Illinois Chicago, 801 S. Paulina St, Chicago, IL 60612, USA

**Keywords:** *Dermanyssus gallinae*, extermination, insecticide, drug residue

## Abstract

A safe and effective method for eradicating poultry red mite (PRM; *Dermanyssus gallinae*) is urgently needed, as existing treatments show a low efficacy or hazardous effects on chickens. We evaluated the efficacy of a combined treatment with ivermectin and allicin (IA) against PRMs in chickens and drug residues in non-target samples. The efficiency of PRM eradication by IA was compared with those of natural acaricides in vitro. Ivermectin (0.25 mg/mL) + allicin (1 mg/mL) (IA compound) was sprayed on isolator housing hens with PRMs. The PRM mortality rate, clinical symptoms, and ivermectin residue in hens were analyzed. IA showed the highest PRM-eradication efficacy among all tested compounds in vitro. The insecticidal rates of IA were 98.7%, 98.4%, 99.4%, and 99.9% at 7, 14, 21, and 28 days of treatment, respectively. After inoculating PRMs, hypersensitivity, itching, and a pale-colored comb were observed in control animals, which were absent in treated hens. No clinical symptoms from IA and ivermectin residues were found in hens. IA effectively exterminated PRMs, demonstrating its potential for industrial use to treat PRMs.

## 1. Introduction

*Dermanyssus gallinae*, widely known as poultry red mite (PRM), poultry mite, red mite, or chicken mite, is a predominant and critical blood-sucking ectoparasite of layer and breeder flocks worldwide. Poultry production and hen health in Europe, Asia, and the Americas have been greatly affected by PRM infestations. Thus, ongoing efforts and strategic measures are needed in these regions to alleviate the negative effects of PRM on the poultry industry. PRM is the most widespread mite species found in birds in Europe [1]. PRMs mainly attack resting chickens at night for a short period (30–60 min) by sucking their blood [2,3]. After the blood meal, PRMs inhabit the crevices of the host skin, where they digest the sucked blood, mate, and lay eggs [1,4]. Nymphs and females suck blood, whereas males only do so occasionally; the larvae do not suck blood [1]. Severe PRM infestation may result in considerable blood loss (anemia) and other diseases caused by transmitted viruses, bacteria, and parasites [5,6,7], possibly leading to host mortality. Other adverse effects of PRM infections in chickens are reduced egg quality because of blood spots, stress, and body-weight reduction [8]. Therefore, PRMs must be eradicated to maintain the health of poultry and prevent gross agroeconomic losses.

Acaricide application is the main approach used to exterminate PRMs, and certain acaricides have been approved globally. The most widely used acaricides are organophosphates, carbamates, amidines, and pyrethroid-based acaricides. However, many acaricides are not recommended against PRMs but are illegally used on poultry farms in various countries [8,9,10,11,12]. For example, the acaricide fipronil is not classified as an “allowed substance” for use as a veterinary medicinal product in food-producing animals and birds [13]. Although some acaricides are effective against PRMs, they also affect non-targets such as humans, poultry, and eggs. Moreover, PRMs have developed resistance to different classes of acaricides in various regions worldwide. For example, manufacturers claim that the acaricide fluralaner greatly reduces red mite populations, and its sales in the field have significantly increased. However, veterinarians treating chickens in the field suggest that resistance to fluralaner has recently emerged. Therefore, several alternative solutions, including biological compounds, essential oils, heat treatments, predator mites, inert dust, intermittent lighting programs, and even vaccines, have been developed [14].

The anthelmintic drug ivermectin (0.5% lotion) can kill head lice (a human ectoparasite) [15]. Garlic has a lethal effect on northern fowl mites [16]; its main component, allicin, is thought to be responsible for its biological activity [17]. We previously reported that a combination of ivermectin and allicin (IA) has potent PRM-eradication effects in vitro [18]. However, the effect of IA was not compared with those of commercially available acaricides, and we did not evaluate whether the IA has adverse effects on hens. Thus, in this study, we compared the PRM-exterminating effect of IA with those of commercially available natural acaricides. We also examined the PRM-eradication effect of IA in a real environment in poultry housing and whether IA has adverse effects and leaves residues in poultry to address public health concerns.

## 2. Results and Discussion

**Figure 1:**
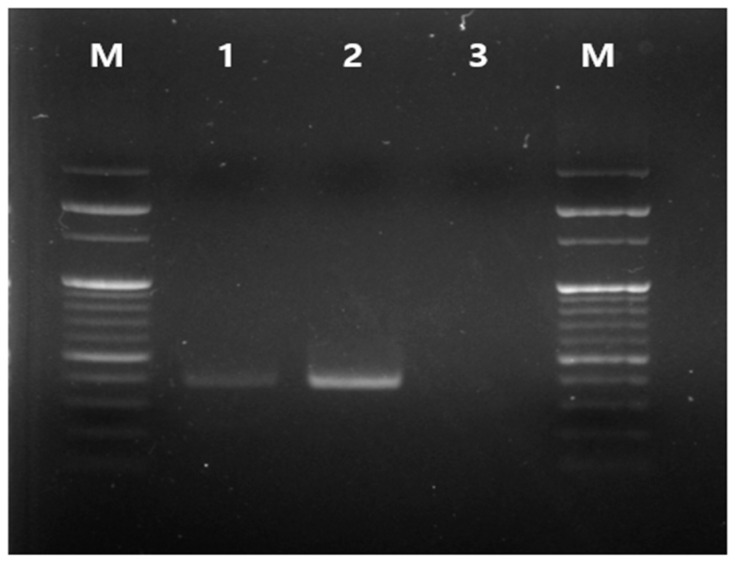
PCR band of poultry red mites (*Dermanyssus gallinae*). M: 100-bp ladder, 1: collected poultry red mites, 2: positive control, 3: negative control.

Chemicals used to control PRMs may adversely affect workers through direct exposure and indirect consumption of pesticide-residue-containing eggs [19]. We aimed to develop a more effective and convenient treatment for exterminating PRMs without harming target/non-target animals and humans by using IA. Ivermectin is an efficient and safe treatment for controlling the growth of PRMs [20]. We previously found that IA has synergistic effects in exterminating PRMs in vitro [18]. In this study, we investigated the efficacy of IA against PRMs in a real-world poultry-housing environment and evaluated whether IA has adverse effects and/or leaves residues in poultry. Collected mites were identified as PRMs using the PCR method shown in Figure 1. The band of collected PRM showed a similar pattern to the positive control.

The PRM-eradication rate of IA was compared with those of natural agents. The corrected eradication rates of clove extract, cypress oil, and shrubby sophora extract (active ingredients of natural acaricides on the market) were 11.7%, 58.3%, and 26.7%, respectively. In contrast, 100% of PRM was eradicated by IA in vitro (Table 1). According to the South Korean Ministry of Food and Drug Safety’s “Efficacy Testing Guidelines for Insecticides for Prevention of Infectious Diseases”, direct sprays cannot be approved unless the eradication rate is greater than 90% after 24 h from the time of application. Therefore, the efficacy of most commercially available natural acaricides, except for that of pyrethrim, is inadequate. Pyrethrum, a pyrethroid insecticide, is a natural substance that was used as a positive control. Although it is an effective insecticide, it can affect the central nervous system of animals and humans, and it is associated with safety issues. IA also showed a 100% PRM-eradication rate in our previous in vitro study [18].

**Figure 2:**
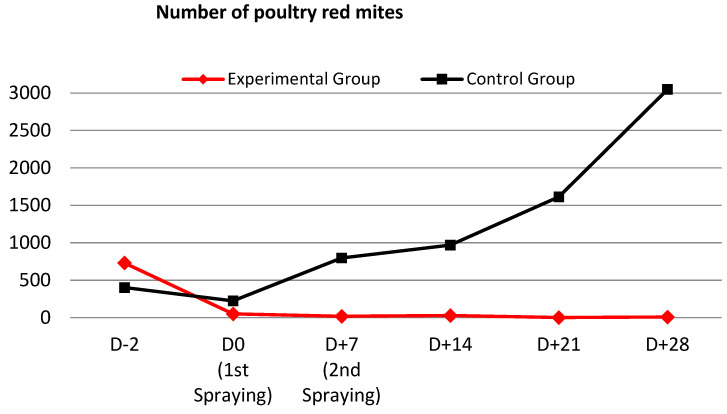
Poultry red mites (*Dermanyssus gallinae*) extermination efficacy by IA in a farm environment in chickens (number of poultry red mites found in the trap).

Based on the in vitro PRM-eradication efficacy of IA, the PRM-extermination effect of IA was evaluated in chickens inside of a chicken cage. We found that 731 and 403 PRMs were captured by the trap at 2 days before the first spraying of IA (Figure 2). The number of captured PRMs gradually decreased over the experimental period, which was the opposite of the results observed in the control animal group on different observation days. At day 28 after the first spraying, 8 and 3049 live PRMs were found in the trap in the IA-treated and control groups, respectively, indicating that IA spray kills PRMs but a larger number of PRMs grew in the control group.

The results of efficacy evaluation (Table 2) showed that the number of PRMs decreased by 93.1% in the experimental group after the first IA spraying (D0), with an efficacy of 87.6%. After the second spraying, at one week after the initial spraying (D + 7), the number of PRMs decreased by 97.4% with a compound efficacy of 98.7%. The overall decrease in PRMs up to 28 days after the initial spraying was 98.9%, with the final efficacy of the compound being 99.9%.

**Figure 3:**
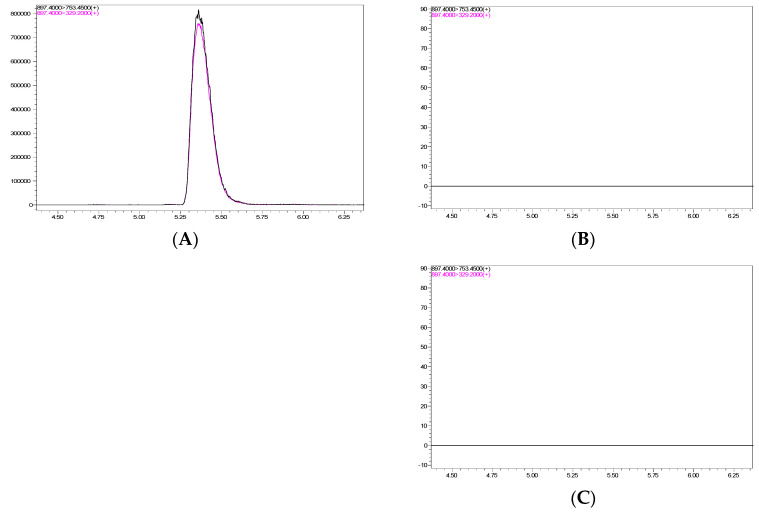
LC–MS/MS analysis results of the blood and egg samples. The chromatogram of (**A**) the ivermectin standard (500 ppb), (**B**) a blood sample at 6 h and (**C**) an egg sample at 24 h. The retention time and mass transition of ivermectin were 5.37 min and 897.4, respectively.

The presence of ivermectin residue in the blood and eggs was detected at 1, 6, 24, 48, and 72 h and 7 days after the second spraying to evaluate its residual effect in chickens. The blood analysis results obtained using liquid chromatography–tandem mass spectrometry (LC–MS/MS) revealed no ivermectin residues in the entire sample (Figure 3). Thus, residues in the edible areas were not tested, as no IA compound residues were detected in the blood samples.

**Figure 4:**
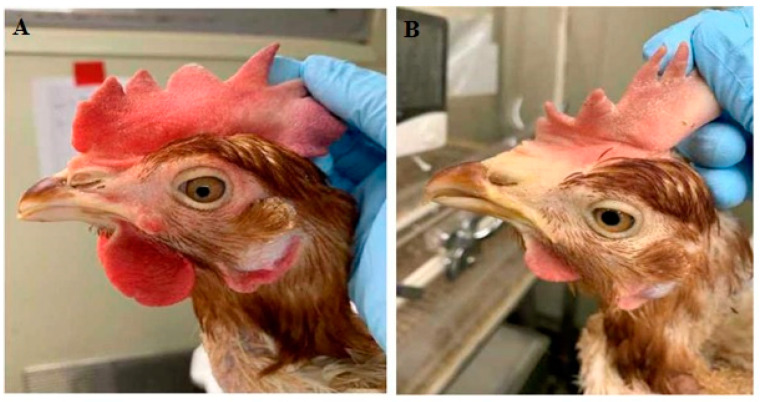
Comb status of chickens in (**A**) the experimental group treated with insecticide and (**B**) the control group not treated with insecticide. Pictures were taken at 5 days after initial spraying of IA.

The average weights of the hens and eggs were measured, but no significant difference was observed between the experimental and control groups. Spraying of IA did not evoke specific clinical symptoms, such as depression or edema, confirming the absence of safety issues. After inoculating the hens with PRMs, abnormal reactions, such as hypersensitivity and itching, were observed, and the comb color was paler in the control group of hens. Hens in the experimental and control groups showed a difference in the comb color after administering IA (Figure 4). The combs of hens in the control group appeared pale in color. However, hens in the experimental group (infected with PRMs and sprayed with IA) showed a usual comb color, demonstrating that PRMs caused stress in control hens but not in hens sprayed with IA. These data indicate that acaricide decreased the number of mites, leading to an alleviation of clinical symptoms in the experimental group.

## 3. Materials and Methods

### 3.1. Samples

Mites were collected from a regional poultry farm (Yangsan, Gyeongsangnam-do), identified as PRMs (*D. gallinae*) using a previously described PCR method [21], and stored at 4 °C until further experiment. DNA was extracted from the red mites using the commercial kit DNeasy Blood and Tissue (Qiagen, Hilden, Germany) for identification. A PCR assay was performed using primers specific for amplification of the mitochondrial 16S rRNA gene of *D. gallinae*. Specific primer sequences used to identify the PRMs were F16 (5′-TGGGTGCTAAGAGAATGGATG-3′) and R16 (5′-CCGGTCTGAACTCAGATCAAG-3′) (accession number L34326), which amplify a 377-bp region. The PRM-eradication effects of various natural agents such as cloves, cypress oil, shrubby sophora, and pyrethrum and the chemical compounds ivermectin and allicin were evaluated. The treatment agents were diluted in polyethylene glycol 400 (PEG400).

### 3.2. In Vitro PRM-Eradication Effect

**Figure 5:**
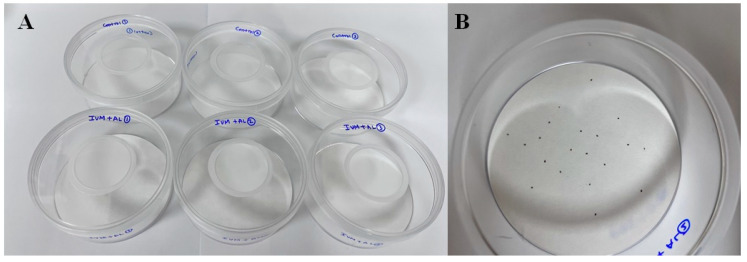
Evaluation of efficacy of IA in vitro. (**A**) Insect culture plates with filter papers inoculated with diluted acaricide and (**B**) inoculation of poultry red mites.

A 90-mm filter paper (100-0011, Advantech, Tokyo, Japan) was placed in each insect-culture dish (ICD), with 1 mL of PEG400 (Sigma Chemical Co., St. Louis, MO, USA) as an untreated control. Cloves (5.6 mg/mL as eugenol, Sigma Chemical Co.), cypress oil (49 mg/mL as dipentene, Sigma Chemical Co.), shrubby sophora (22.5 mg/mL as matrine, Sigma Chemical Co.), pyrethrins (1 mg/mL, Sigma Chemical Co.), ivermectin (1.0 mg/mL) + allicin (0.5 mg/mL), and ivermectin (0.25 mg/mL) + allicin (1.0 mg/mL) were applied to the filter papers in different ICDs using a micropipette. The concentrations of these active ingredients were selected based on their effective concentrations in commercially available products. On a working bench, the filter papers were left to soak the drug solution for 30 min and then partially dried. PRMs (20 individuals) were transferred to each ICD (Figure 5). The PRMs and drug-containing ICDs were stored in insulated foam containers at 25 °C and 70% humidity, and the death rates of PRMs were recorded after 2, 24, and 48 h of treatment. The described method is based on the “contact filter paper susceptibility and resistance test” outlined in the guidelines for insecticide efficacy testing [22].

### 3.3. In Vivo PRM-Eradication Effect

**Figure 6:**
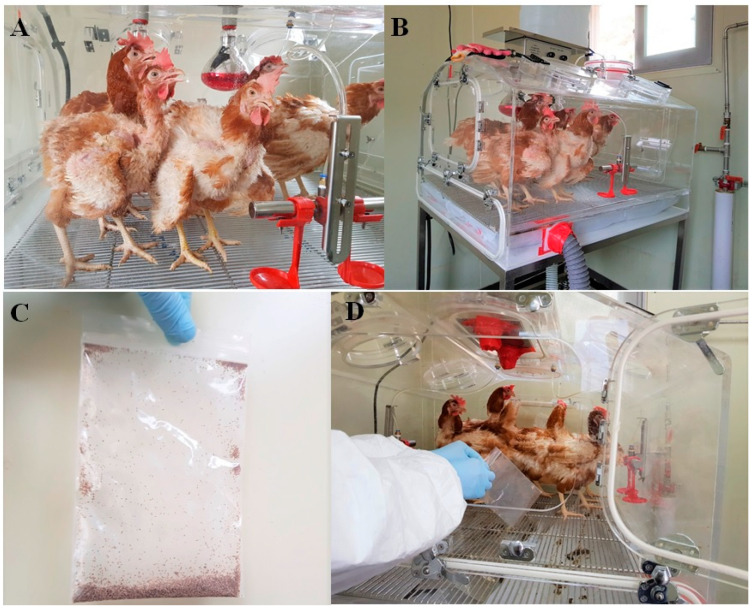
Evaluation of efficacy of IA compound on the chickens. (**A**) Sample animals (hens). (**B**) Raising hens in the isolator. (**C**) Poultry red mite sample. (**D**) Inoculating poultry red mites into the isolator.

Based on the in vitro PRM-eradication effect, the most potent combination of ivermectin 0.25 mg/mL + allicin 1 mg/mL (IA compound) was selected for in vivo efficacy testing in chickens. Ten hens aged 30 weeks were equally divided into an experimental group (treated with the drug) and a control group (not treated with the drug). Hens in the experimental and control groups were raised in designated isolators. Subsequently, 1000 PRMs stored in a freezer were placed in each isolator to infect the hens (Figure 6). The transmission of PRMs and resulting clinical symptoms (stressful behavior; hypersensitivity reaction, itching) of the chickens were observed for four weeks after the initial infection. The corrugated cardboard method was used from the third week after the initial infection to check for contamination levels of PRMs. After confirming the infestation level of the PRMs had reached the most severe stage commonly used in field treatments, with approximately 500 mites captured using the cardboard trap method, IA was sprayed (157.3 mL/m^2^) into the isolator using a sprayer without touching the hens, feed, or drinking water. The study involved a total of two spray applications (D0, D + 7). The first application of IA (157.3 mL/m^2^) was followed by a second application at the same concentration one week after the initial treatment. Control chickens were managed in the same manner, with distilled water sprayed at the same volume as used for treated chickens.

A previously reported corrugated cardboard trapping method [23] was used to determine the number of PRMs. The corrugated cardboard trap was installed before spraying; on the day of spraying; and at 7, 14, 21, and 28 days after spraying, and the number of PRMs remaining was determined. The number of PRMs was recorded two days before spraying the drug (D-2). During the efficacy test, corrugated cardboard traps were installed immediately after spraying of the drug (D0) and at subsequent seven-day intervals to determine the number of PRMs in the experimental and control groups. The Hender–Tilton equation [24,25], described in the guidelines for insecticide efficacy tests provided by the Guidelines for the Safety and Efficacy of Veterinary Drugs [22], was utilized to calculate the insecticidal rate (%). The effect was considered successful if the rate exceeded 95%.

$$\mathrm{Insecticidal}\;\mathrm{rate}=\left[1-\left(\frac{N\;\mathrm{in}\;\mathrm{control}\;\mathrm{group}\;\mathrm{before}\;\mathrm{treatment}\;\times \;N\;\mathrm{in}\;\mathrm{treatment}\;\mathrm{group}\;\mathrm{after}\;\mathrm{treatment}\;}{N\;\mathrm{in}\;\mathrm{control}\;\mathrm{group}\;\mathrm{after}\;\mathrm{treatment}\;\times \;N\;\mathrm{in}\;\mathrm{treatment}\;\mathrm{group}\;\mathrm{before}\;\mathrm{treatment}}\;\right)\right]\times 100\;\left(\%\right)$$

where N is the insect population

### 3.4. Evaluation of Ivermectin Residue in Chickens

According to Article 6 (Sample Collection) of Appendix 6 (Residue Testing Methods of Veterinary Medicinal Products) of the Guidance for Residues of Veterinary Medicinal Products (No. 2016-23, issued on 9 March 2016, by the Animal and Plant Quarantine Agency) [26], drugs used in farming environments that can be absorbed by livestock should be analyzed from other edible areas only if the drug residue is detected in the blood, eggs, and oil. The presence of ivermectin residue in the chicken blood and eggs was evaluated to determine its residual effect in chickens. Chicken blood samples were collected before spraying the drug, as well as at 1, 6, 24, 48, and 72 h and 7 days after the second round of spraying. Egg samples were collected whenever they were found during the experiment. LC–MS/MS was performed to analyze residual ivermectin in the blood and egg samples. The LC–MS/MS system was equipped with a high-performance liquid chromatography (2695 Separations Module, Waters Corporation, Milford, MA, USA), mass spectrometer detector (Quattro Micro API, Waters Corporation), and waters X-Bridge C18 (2.1 × 150 mm, 3.5 μm) column (temperature at 40 °C) for chromatographic separation. The mobile phase was a mixture of (A) 0.1% formic acid and 5 mM ammonium formate in distilled water and (B) 0.1% formic acid and 5 mM ammonium formate in acetonitrile, where the ratio of “A” and “B” was different and maintained in a gradient flow. Initially, 20% of B was applied from 0 to 1.5 min. The ratio of B was gradually increased to 100% from 1.5 to 3.0 min and maintained at this ratio up to 7.0 min. From 7.0 to 8.0 min, the ratio of B was returned to 20%. The same composition was maintained until the end of the run (up to 10 min). The flow rate of the mobile phase was 0.4 mL/min. The volume of each injection was 1 μL, and the run time for a single injection was 10 min.

## 4. Conclusions

The demonstrated efficacy of IA treatment in exterminating PRMs under real-life poultry-farm conditions shows potential for use on farms. Our data reveal that IA treatment significantly reduced PRM populations in chicken cages without causing clinical symptoms or adverse effects in hens as the non-target subjects. Moreover, ivermectin residues were not detected in the blood or eggs of treated chickens, ensuring safety for consumption. The use of the IA combination to control PRMs without causing harm to the hens or leaving residues emphasizes its potential for use in the poultry industry. Our results provide insights for policy makers and stakeholders in making informed decisions on the adoption of IA for treating PRM in industrial settings.

## Figures and Tables

**Table 1:** In vitro poultry red mites (*Dermanyssus gallinae*) extermination efficacy by IA and other commercially available natural acaricides.

Group	Plate No.	Number of Dead Red Mites		^(a)^ Mortality Rate (%)	Average (*n* = 3)	Standard Deviation	^(b)^ Corrected Eradication Rate (%)
		24 h	48 h				
Untreated control (PEG400)	1	0	0	0	0	-	0
	2	0	0	0			
	3	0	0	0			
Clove extract	1	3	3	15	20	8.7	20
	2	1	3	15			
	3	3	6	30			
Cypress oil	1	6	11	55	68.3	15.3	68.3
	2	13	13	65			
	3	16	17	85			
Shrurry sophora extract	1	7	13	65	40	27.8	40
	2	9	9	45			
	3	0	2	10			
Ivermectin (1.0 mg/mL) + allicin (0.5 mg/mL)	1	20	20	100	100.0	-	100.0
	2	20	20	100			
	3	20	20	100			
Ivermectin (0.25 mg/mL) + allicin (1.0 mg/mL)	1	20	20	100	100.0	-	100.0
	2	20	20	100			
	3	20	20	100			
Positive control (pyrethrin)	1	20	20	100	100.0	-	100.0
	2	20	20	100			
	3	20	20	100			

^(a)^ The 24-h mortality rate (initial number of mites: 20): (killed mites/initial mites) × 100. ^(b)^ The 24-h corrected eradication rate: [(mortality rate in experimental group − mortality rate in control group)/(100 − mortality rate in control group)] × 100.

**Table 2:** Poultry red mite (*Dermanyssus gallinae*) extermination efficacy (reduction rate, %) by IA in a farm environment in chickens, using the Hender–Tilton equation.

Class	D0 (After Initial Spraying)	D + 7 (Second Spraying)	D + 14	D + 21	D + 28
Experimental group	93.1%	97.4%	96.1%	99.7%	98.9%
Control group	44.0%	−97.8%	−140.6%	−300.6%	−656.6%
IA efficacy	87.6%	98.7%	98.4%	99.4%	99.9%

## Data Availability

Not applicable.
